# Biofilm-driven multi-stage anaerobic-aerobic process for high-strength petrochemical wastewater treatment

**DOI:** 10.3389/fmicb.2026.1778614

**Published:** 2026-02-25

**Authors:** Dadi Zhao, Guoqiang Ma, Guoyi Li, Chuanguo Zhang

**Affiliations:** 1 Key Laboratory of Environmental Protection Technology on Water Transport, Tianjin Research Institute for Water Transport Engineering, M.O.T., Tianjin, China; 2 Environmental Technology Development of TIWTE (Tianjin) Co., Ltd., Tianjin, China

**Keywords:** biological contact oxidation, coagulation-Fenton, hydrolysis-acidification, multi-stage anaerobic-aerobic coupling, petrochemical wastewater

## Abstract

High-strength petrochemical wastewater typically shows poor biodegradability, making stable compliance difficult with biological treatment alone. In this study, an integrated train combining coagulation–Fenton oxidation pretreatment with a biofilm-driven multi-stage anaerobic–aerobic process was developed. The Fenton pretreatment was optimized by response surface methodology, and the downstream system comprised an anaerobic biofilter, multi-stage biological contact oxidation, followed by hydrolysis–acidification/contact oxidation and clarification. Results indicated that the Initial pH was the most influential factor for Fenton performance. Under optimized conditions (pH 2.20, H₂O₂ dosage 4.5 mL/L, H₂O₂/Fe^2+^ molar ratio 20), pretreatment achieved 51.9% COD removal. At steady operation (Day 28), overall COD decreased from 3740 mg/L to 239.2 mg/L (93.6% cumulative removal). Anaerobic biofilter provided stable COD removal of 20.2–23.5% with an optimal temperature window of 25–35°C, while the multi-stage biological contact oxidation maintained 64.1–80.0% COD removal and was buffered under higher loading by extending reaction time/increasing hydraulic retention time. Biofilm stability was supported by MLSS of 4,151 mg/L and SVI of 75.9 mL/g in the multi-stage reactor (Day 30). Overall, coagulation–Fenton served as an influent-shaping module, complementing the anaerobic–aerobic biofilm process to achieve robust removal of high-strength refractory organics.

## 1 Introduction

Petrochemical and fine chemical wastewaters are characterized by high pollutant concentrations, complex compositions, and poor biodegradability due to diverse raw materials and intricate reaction mechanisms, posing severe challenges to current industrial water treatment (Guo et al., 2024). These effluents, typically originating from polymerization, separation and washing processes, contain substantial amounts of unreacted monomers and by-products, including long-chain polymers, graft copolymers, and various surfactants. The presence of stable molecular structures often carrying refractory functional groups such as ether bonds, cyano groups, and amino groups—contributes to extremely high chemical oxygen demand (COD) and low bioavailability (Chan et al., 2009; Puyol et al., 2017; Wang et al., 2020).

Furthermore, the extensive use of nitrogenous compounds, such as organic amines and acrylonitrile, results in effluents with concurrent high organic carbon and nitrogen loads (“high-C/high-N”) (Wei et al., 2021; Cui et al., 2025b; Zhao et al., 2025). This characteristic, combined with the inherent toxicity of polymeric compounds, significantly inhibits microbial metabolic activity in conventional activated sludge systems, leading to process instability and discharge non-compliance (Barancheshme and Munir, 2018; Uluseker et al., 2021; Cui et al., 2025a; Cui et al., 2025c). In practice, the “high-C” characteristic is operationally manifested as elevated COD, and COD reduction is the prerequisite for maintaining stable anaerobic hydrolysis and robust aerobic biofilm mineralization under fluctuating influent conditions. Moreover, COD is the most widely reported and regulation-relevant indicator for industrial wastewater compliance and cross-study benchmarking, enabling clear comparison of stage-wise contributions and integrated treatment efficiency.

To mitigate these challenges, physicochemical methods such as Advanced Oxidation Processes have been employed to degrade refractory organics (Oller et al., 2011). Among these, Fenton reagent (H₂O₂/Fe^2+^) is recognized for its strong oxidizing capability, effectively degrading organic wastewaters containing phenols and other recalcitrant compounds that are resistant to general chemical oxidants (Padoley et al., 2011; Cheng et al., 2022). However, while Advanced Oxidation Processes are effective, their high operational costs and chemical consumption limit their application as a standalone solution for full mineralization of high-strength wastewater.

A more economically viable and technically robust strategy involves coupling chemical pretreatment with biological processes (Ding et al., 2016; Ding et al., 2018). In this approach, Fenton oxidation serves as the “detoxification” and “structural modification” step, cleaving long-chain macromolecules into smaller, biodegradable volatile fatty acids or less toxic intermediates (Wu et al., 2018). This pretreatment significantly improves the ratio of biochemical oxygen demand to COD (B/C), creating a favorable environment for subsequent biological treatment. Following pretreatment, selecting an appropriate biological process is critical. Conventional suspended-growth anaerobic reactors (e.g., UASB) often suffer from biomass washout and granulation difficulties when treating wastewater containing specific toxic compounds (Ding et al., 2016; Chu et al., 2021; Huang et al., 2021). In contrast, biofilm-based technologies offer distinct advantages. Biofilms attached to carrier media possess longer solids retention times (SRT) and stratified microenvironments, which facilitate the retention of slow-growing hydrolytic bacteria and nitrifiers while offering superior resistance to shock loading compared to suspended sludge systems (Wang et al., 2021).

In this study, an integrated treatment train combining Coagulation-Fenton pretreatment with an AF and a Multi-stage Biological Contact Oxidation (MBCO) process was developed for high-strength petrochemical wastewater containing refractory organics. The coagulation–Fenton step was implemented as an influent-conditioning module to partially oxidize recalcitrant organics, thereby providing a more stable feed for downstream bioprocesses. The AF unit, operated with attached anaerobic biofilms, served as a buffering and pre-conversion stage, while the MBCO unit provided the primary aerobic mineralization under a staged (plug-flow-like) configuration. This work optimized key Fenton operating factors using Response Surface Methodology (RSM) and subsequently evaluated the startup performance and biofilm establishment of the coupled AF–MBCO system treating the pretreated effluent. In addition, system performance under steady operation was assessed, with emphasis on stage-wise contributions and the temperature dependence of anaerobic hydrolysis in the AF. Overall, the study provides engineering-relevant operating parameters for coupling chemical oxidation with biofilm-based biological treatment to achieve robust removal of refractory organic loads.

## 2 Materials and methods

### 2.1 Coagulation experiments

Coagulation experiments were conducted using a six-gang stirrer. A volume of 600 mL of petrochemical wastewater was transferred into the reaction beaker, followed by the addition of 27 mg of coagulant to achieve a final dosage of 45 mg/L. Upon addition of the chemical, the mixing protocol was initiated: a rapid mixing stage was performed at 300 r/min for 1 min to ensure rapid dispersion of the coagulant, followed by a slow mixing stage at 150 r/min for 20 min to promote flocculation. After the mixing process, the paddles were withdrawn, and the samples were allowed to settle statically for 30 min.

### 2.2 Fenton oxidation pretreatment experiments

The Fenton oxidation pretreatment experiments were conducted in batch mode using beakers with an effective working volume of 500 mL. During the process, the reaction temperature was maintained at 20–25 °C, and the solution was mixed via a mechanical stirrer at a speed of 90–120 r/min for a reaction duration of 60 min. To optimize the pretreatment efficiency for alleviating the load on the subsequent biological system, RSM was employed. Based on preliminary single-factor experiments, initial pH (*X_1_*), H₂O₂ dosage (*X_2_*), and H₂O₂/Fe^2+^ molar ratio (*X_3_*) were selected as the three independent variables, with COD removal efficiency (Y) as the response variable. A three-factor, three-level experimental design was generated. As shown in Table 1, the coding and levels of the factors were established as follows: initial pH (1.5, 3.0, 4.5), H₂O₂ dosage (2, 4, 6 mL/L), and H₂O₂/Fe^2+^ molar ratio (10, 20, 30) (Table 1). The experimental data were fitted using a quadratic polynomial regression equation to determine the optimal operating parameters and analyze the interactions among the factors.

**Table 1:** Factors and levels used in the response surface design for Fenton oxidation.

*X_j_*	Units	Coded factors *X_1_, X_2_, X_3_*		
		−1	0	1
*X_1_ =* initial pH	-	1.5	3.0	4.5
*X_2_ =* H_2_O_2_ dosage	mL/L	2	4	6
*X_3_ =* H_2_O_2_/Fe^2+^ (molar ratio)	-	10	20	30

### 2.3 Lab-scale simulation apparatus

After coagulation, the effluent was introduced into the downstream treatment units. A dedicated lab-scale simulation apparatus was employed to conduct laboratory experiments and assess stage-specific performance within the biological treatment train.

**Figure 1:**
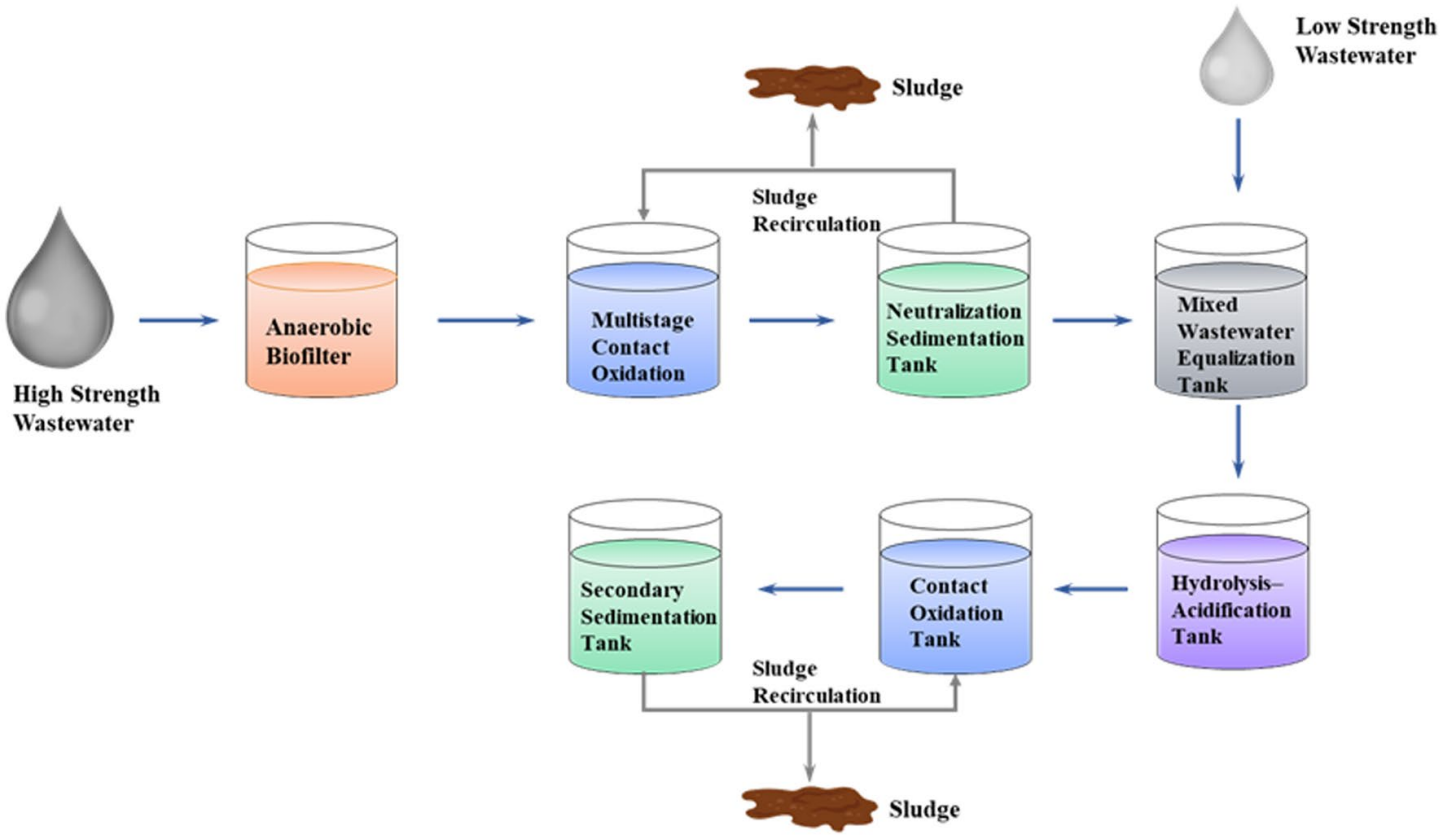
Flowchart of the laboratory-scale simulation experiment for concentrate recirculation.

**Figure 2:**
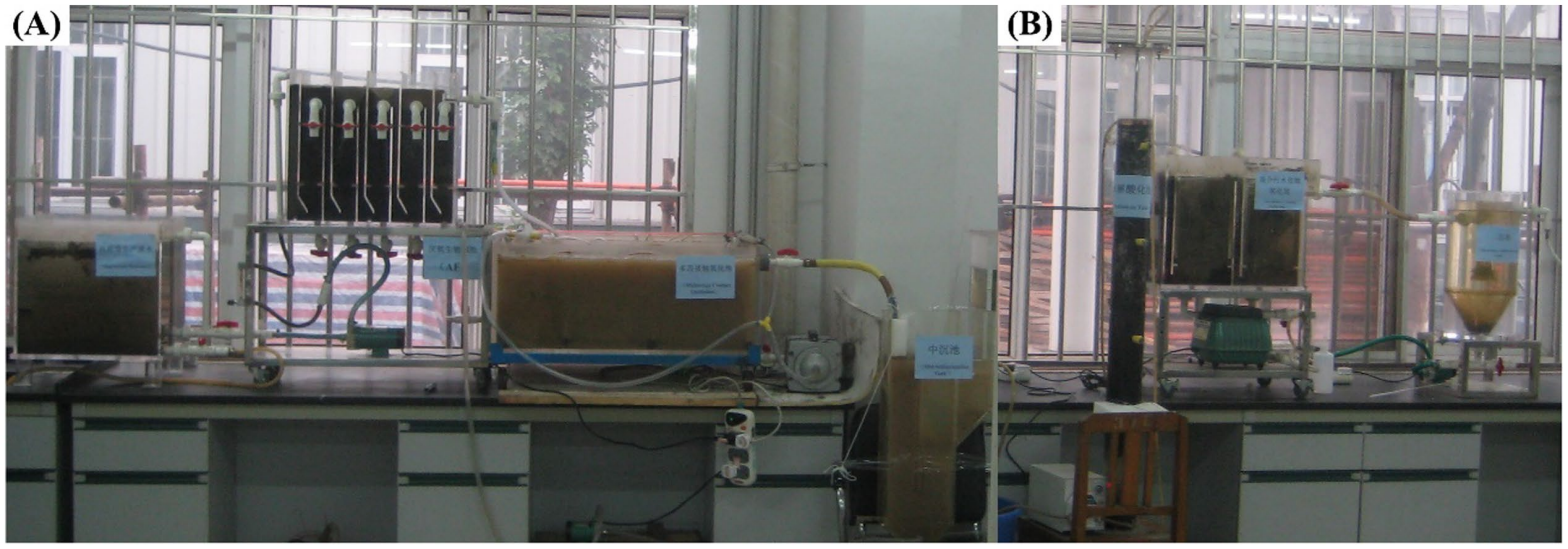
Schematic of the laboratory-scale experimental setup for concentrate treatment. **(A)** AF, followed by multi-stage oxidation and final neutralization–precipitation process; **(B)** hydrolysis acidification followed by contact oxidation and secondary sedimentation.

In this process, high-strength petrochemical wastewater undergoes Coagulation-Fenton pretreatment before entering the unified biochemical system. Initially, the high-concentration wastewater flows into an AF for pre-degradation, followed by a multi-stage contact oxidation tank for stepwise aerobic treatment, and then enters an intermediate sedimentation tank for solid–liquid separation (Figure 1). The effluent is then combined with diluted low-concentration wastewater in a mixing equalization tank. After homogenization, the mixture enters a hydrolysis acidification tank to further enhance the biodegradability of refractory organic matter. Subsequently, the hydrolyzed wastewater is transferred to a contact oxidation tank for advanced aerobic treatment, with the final effluent flowing into a secondary sedimentation tank for separation (Figure 2). A portion of the sludge from the secondary sedimentation tank is recirculated to the contact oxidation tank to maintain biomass, while the excess sludge is discharged for unified disposal. Ultimately, the entire system achieves synergistic and stable treatment of wastewater from different sources through the pathway of “pretreatment – hydrolysis acidification – contact oxidation – sedimentation separation – sludge return.”

### 2.4 Sludge cultivation and microbial acclimation

Prior to the formal start-up of the system, the seed sludge underwent a cultivation and acclimation process to adapt to the specific wastewater matrix. During this phase, the Sludge Volume Index (SVI) was employed as the critical control parameter to evaluate the settling properties and biological activity of the activated sludge. Monitoring results indicated a significant improvement in settling performance following acclimation, with the 30-min Sludge Volume Index (SVI₃₀) decreasing from an initial value of 113.1 mL/g in the raw sludge to 70.9 mL/g in the acclimated sludge. The acclimated sludge exhibited excellent flocculation and settling characteristics, providing a robust basis for the subsequent reactor inoculation and biofilm formation.

### 2.5 Biofilm attachment

The biofilm formation and system start-up were initiated using a combined carrier pre-soaking and “stuffing aeration” (batch aeration) method. Initially, the carrier media, consisting of CLS-I type plastic packing and fibrous packing, were immersed in high-strength wastewater for approximately 1 week. This pretreatment aimed to modify the surface properties of the carriers and establish a microenvironment favorable for initial microbial attachment and growth. Subsequently, 5 L of the acclimated seed sludge (characterized by an MLSS of 4.315 g/L, SV of 30.6%, SVI of 70.9 mL/g, and a yellowish-brown appearance) and 5 L of the pre-soaked carriers were introduced into the aerobic reactor. The reactor was filled with high-strength wastewater and subjected to stuffing aeration for 24 h at an aeration rate of 24 L/h without influent or effluent flow. Following the aeration phase, the supernatant was decanted, and the reactor was refilled with fresh high-strength wastewater. This batch aeration cycle was repeated three consecutive times to facilitate the rapid formation and stable immobilization of biomass on the carrier surfaces (Nicolella et al., 2000).

### 2.6 Quantification of biofilm biomass

Unlike suspended cultures, the quantification of sessile biomass is challenged by its fixation on carrier surfaces; therefore, detachment prior to analysis is a prerequisite. Common validated techniques include mechanical shearing, sonication, and chemical-assisted sonication. In this study, a combined mechanical and ultrasonic detachment method was adopted.

Specifically, a defined volume of carriers was sampled (in duplicate for reproducibility) and placed in a clean container with a specific volume of distilled water. The loosely attached biofilm was first removed via manual scrubbing (mechanical detachment). Subsequently, the carriers were treated in an ultrasonic cleaner to dislodge the firmly attached biomass and that within the internal pores. The resulting suspension was filtered, dried, and weighed to determine the biomass dry weight.

## 3 Results

### 3.1 Coagulation–Fenton pretreatment and parameter optimization

To determine the optimal operating parameters for Fenton oxidation, a RSM study based on the Box–Behnken design (BBD) was conducted (Nair et al., 2014). Initial pH (X_1_), H₂O₂ dosage (X_2_), and H₂O₂/Fe^2+^ molar ratio (X_3_) were selected as independent variables, with COD removal efficiency (Y) as the response. The experimental design matrix, along with the observed and predicted COD removal values, is presented in Table 2. The experimental data were analyzed to fit a quadratic polynomial regression model.


$$\begin{array}{l} Y=51.30-9.95X_{1}+4.36X_{2}+0.64X_{3}+2.97X_{1}X_{2}+\\ 0.025X_{1}X_{3}+0.5X_{2}X_{3}-8.9X_{1}^{2}-5.73X_{2}^{2}-4.97X_{3}^{2} \end{array}$$

**Table 2:** Response surface model predictions and experimental validation for the Fenton oxidation process.

No.	1	2	3	4	5	6	7	8	9	10	11	12	13	14	15	16	17
*X*_1_	0	−1	0	1	0	−1	0	0	0	1	0	−1	1	0	1	−1	0
*X*_2_	0	−1	0	0	−1	0	1	0	−1	−1	1	0	0	0	1	1	0
*X*_3_	0	0	0	−1	-1	-1	1	0	1	0	-1	1	1	0	0	0	0
COD removal (%) Actual	51.9	44.1	50.8	26.6	36.5	47.5	45.7	51.0	37.3	19.2	42.9	48.2	27.4	51.3	35.2	48.2	51.5
COD removal (%) Predicted	51.3	45.2	51.3	26.8	36.1	46.8	46.1	51.3	36.4	19.4	43.8	48.0	28.1	51.3	34.1	48.0	51.3

As shown in Table 3, the Analysis of Variance (ANOVA) results demonstrated that the quadratic regression model was statistically robust and provided a high goodness of fit (Li et al., 2013). The overall model was highly significant (*F* = 190.84, *p* < 0.0001), indicating that the selected factors explained the response variation effectively. The model also showed strong explanatory power with R^2^ = 0.9919 and adjusted R^2^ = 0.9815, and a relatively small residual mean square (MS_res = 0.94), suggesting limited dispersion of experimental data around the fitted surface. However, the lack-of-fit test was significant (*F* = 10.57, *p* = 0.0226), implying that a systematic deviation remained beyond the pure experimental error; this was consistent with the small pure error (MS_pure error = 0.19), which increases the sensitivity of the lack-of-fit test. Therefore, while the model is suitable for trend analysis and parameter optimization within the experimental design space, predictive use should be restricted to the tested factor ranges, and further residual diagnostics and additional runs near critical regions are recommended to reduce lack of fit.

**Table 3:** Analysis of variance (ANOVA) for the regression model.

Source	Sum of squares (SS)	Degrees of freedom (df)	Mean square (MS)	*F* value	Prob > F
Model	1621.26	9	180.14	190.84	<0.0001
Residual	6.61	7	0.94	/	/
Lack of fit	5.87	3	1.96	10.57	0.0226
Pure error	0.74	4	0.19	/	/

**Figure 3:**
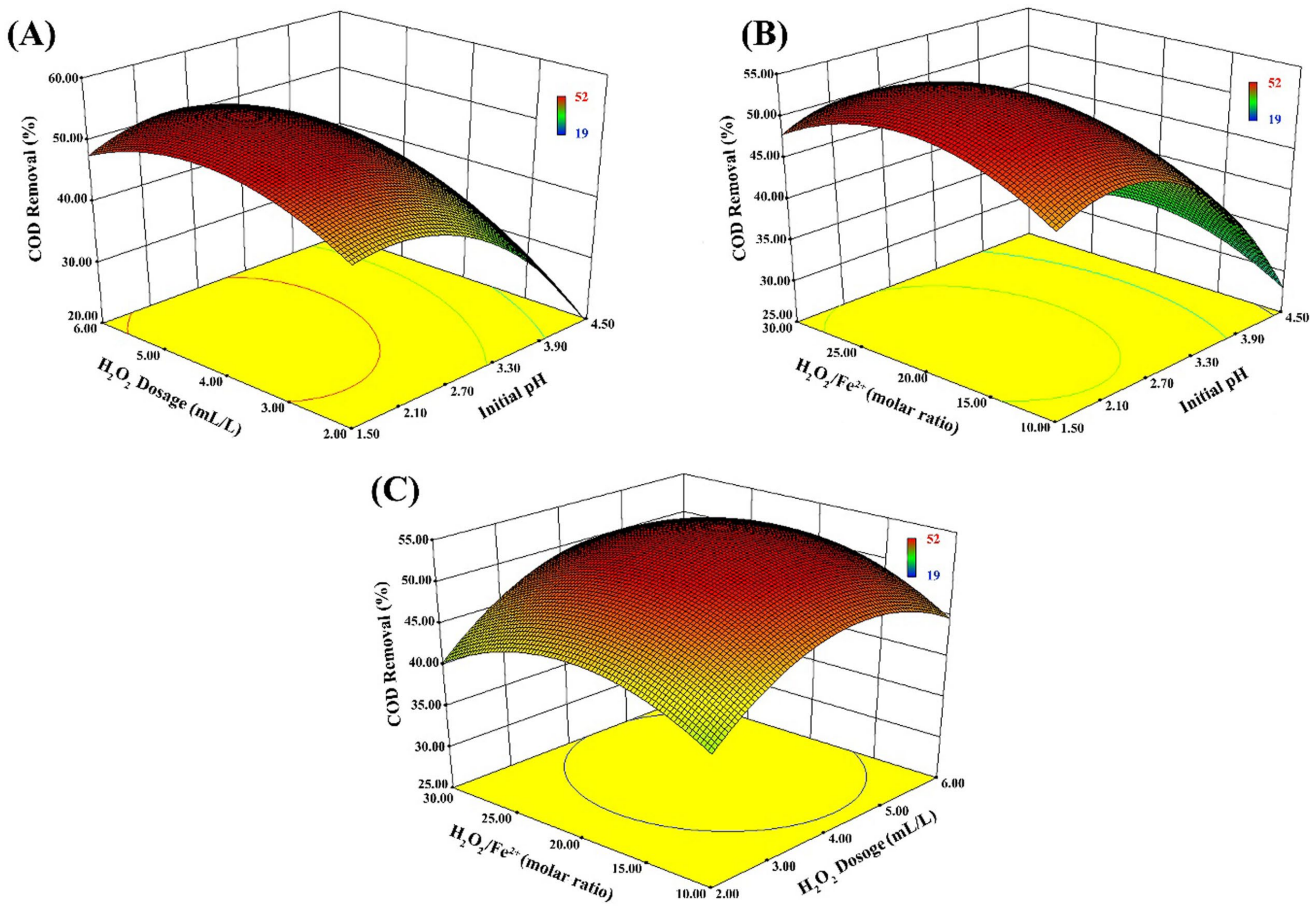
Response surface plots showing the effects of key Fenton operating variables on the predicted COD removal. **(A)** Interactive effect of H_2_O_2_ dosage and initial pH at a fixed H_2_O_2_/Fe^2+^ molar ratio of 20; **(B)** Interactive effect of H_2_O_2_/Fe^2+^ molar ratio and initial pH at a fixed H_2_O_2_ dosage of 4.5; **(C)** Interactive effect of H_2_O_2_ dosage and H_2_O_2_/Fe^2+^ molar ratio at a fixed initial pH of 2.2.

Figure 3 illustrates the 3D response surface plots, visually depicting the interactive effects of the variables on COD removal. The convex shape of the response surfaces indicates the existence of an optimal region within the experimental range. By solving the regression equation, the software predicted the optimal theoretical parameters to be: initial pH = 2.23, H₂O₂ dosage = 4.5 mL/L, and H₂O₂/Fe^2+^ molar ratio = 20.75. Under these conditions, the maximum predicted COD removal efficiency was 54.4%.

For practical feasibility in engineering applications, the operating parameters were adjusted to: initial pH = 2.20, H₂O₂ dosage = 4.5 mL/L, and H₂O₂/Fe^2+^ molar ratio = 20. Under these modified conditions, triplicate verification experiments were conducted, yielding an average COD removal efficiency of 51.9% (with a concurrent oil removal rate of 62.0%). The experimental result aligns closely with the model’s predicted value of 53.7%, confirming the accuracy and validity of the model for optimizing the Fenton pretreatment process. This effective pretreatment step significantly reduced the organic load, creating favorable influent conditions for the subsequent biological treatment system.

### 3.2 Treatment performance of the AF on petrochemical wastewater

**Figure 4:**
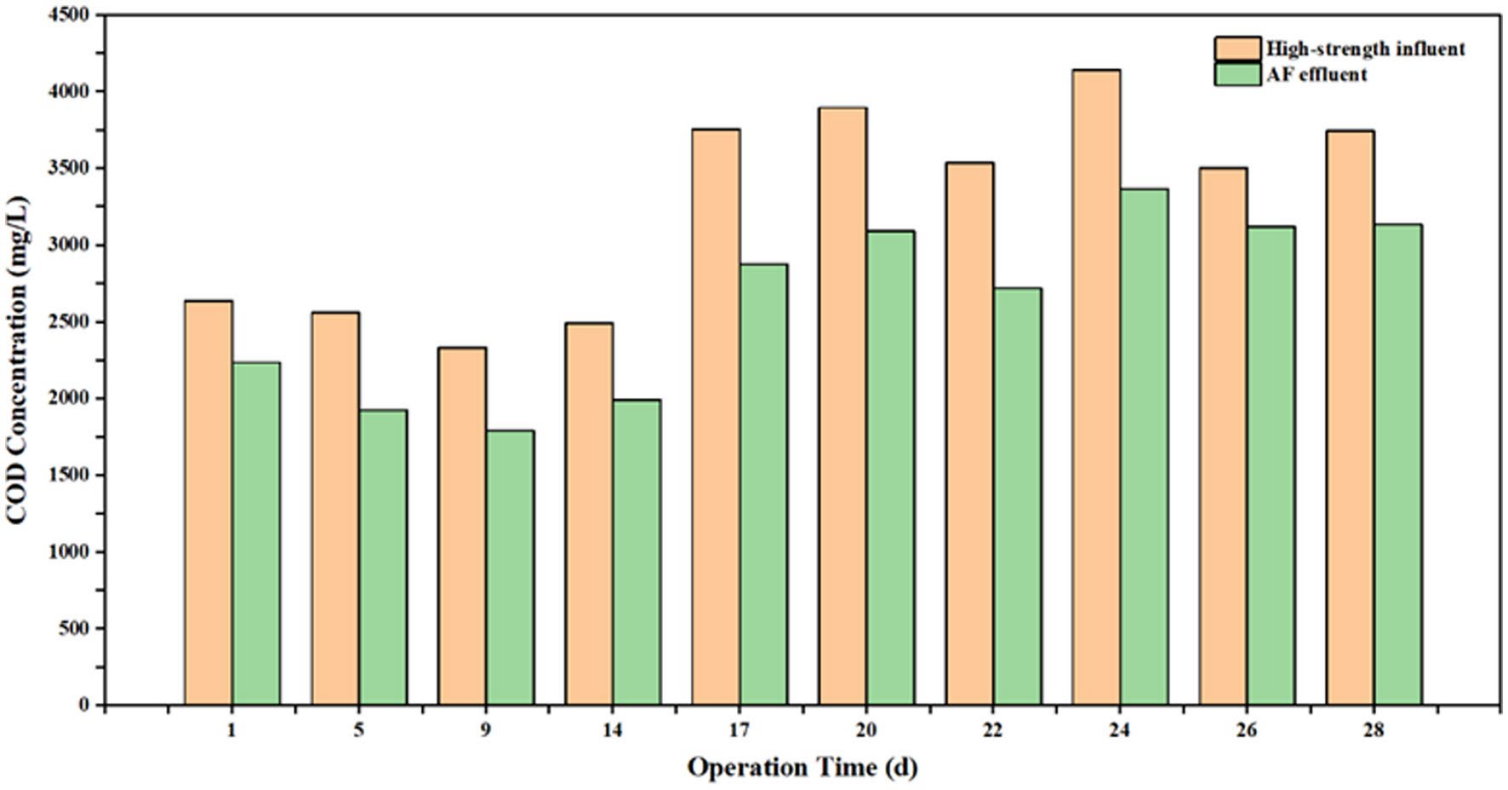
Variations of influent and effluent COD concentrations in the AF reactor over the operational period.

**Figure 5:**
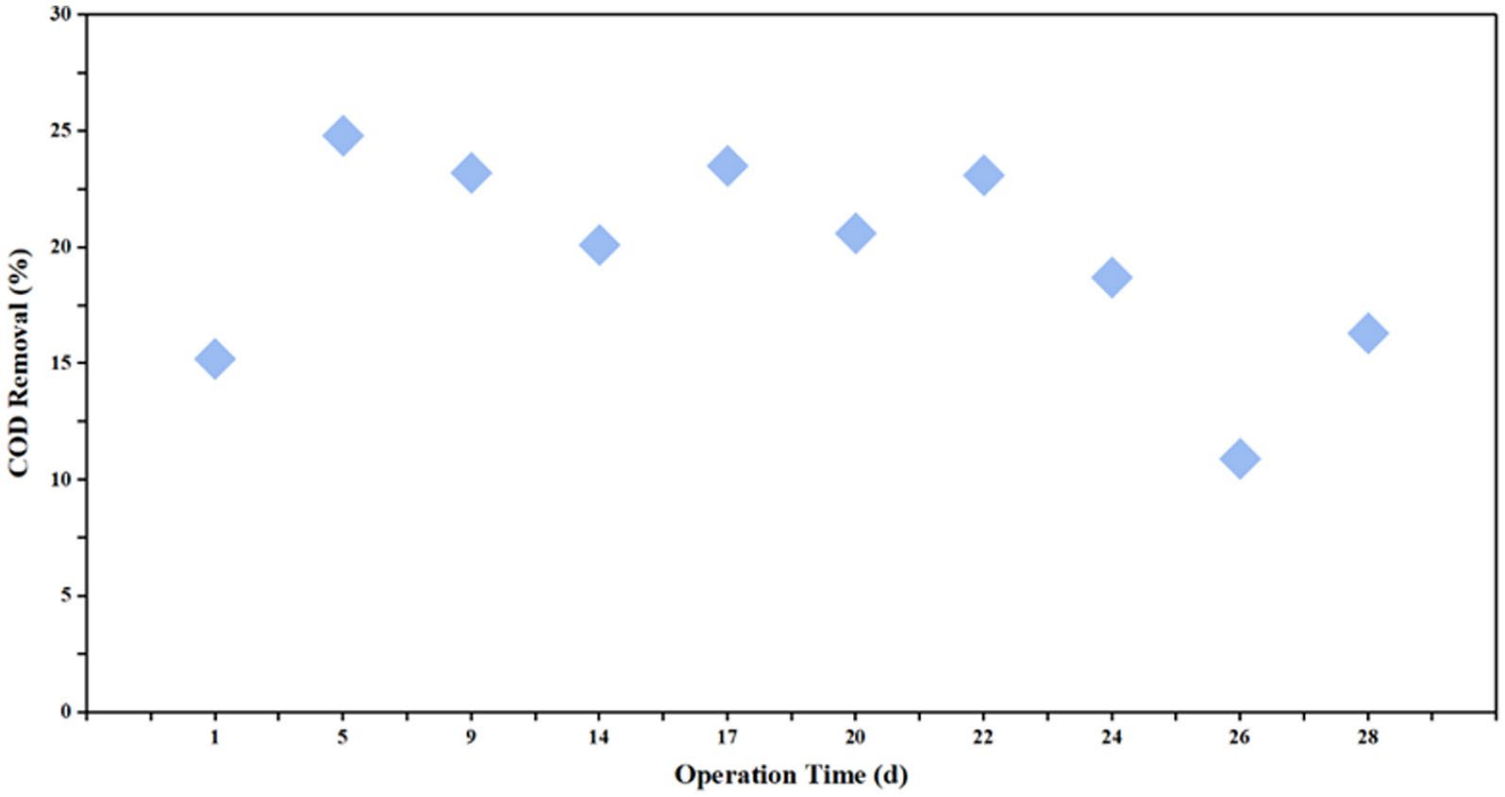
COD removal efficiency of the AF reactor during the operational period.

The COD removal performance of the AF reactor varied throughout the start-up and operational phases (Figures 4, 5). During the initial stage, the removal efficiency was relatively low, standing at only 15.3%. By Day 5, the efficiency increased to 25% and subsequently stabilized within the range of 20.2–23.5%. However, a notable decline was observed on Day 24, with the efficiency dropping to 18.7%, followed by a further decrease to 10.9% on Day 26. After ruling out fluctuations in influent salinity, this performance deterioration was attributed to ambient temperature variations, specifically the significant diurnal temperature difference observed during this period. These findings suggest that the AF reactor possesses an optimal temperature range (25–35 °C in this study) for maximizing organic degradation capacity; deviations from this range adversely affect removal efficiency. Consequently, implementing effective thermal insulation for reactor structures is critical in full-scale engineering applications.

### 3.3 Performance of the multi-stage biological contact oxidation reactor

**Figure 6:**
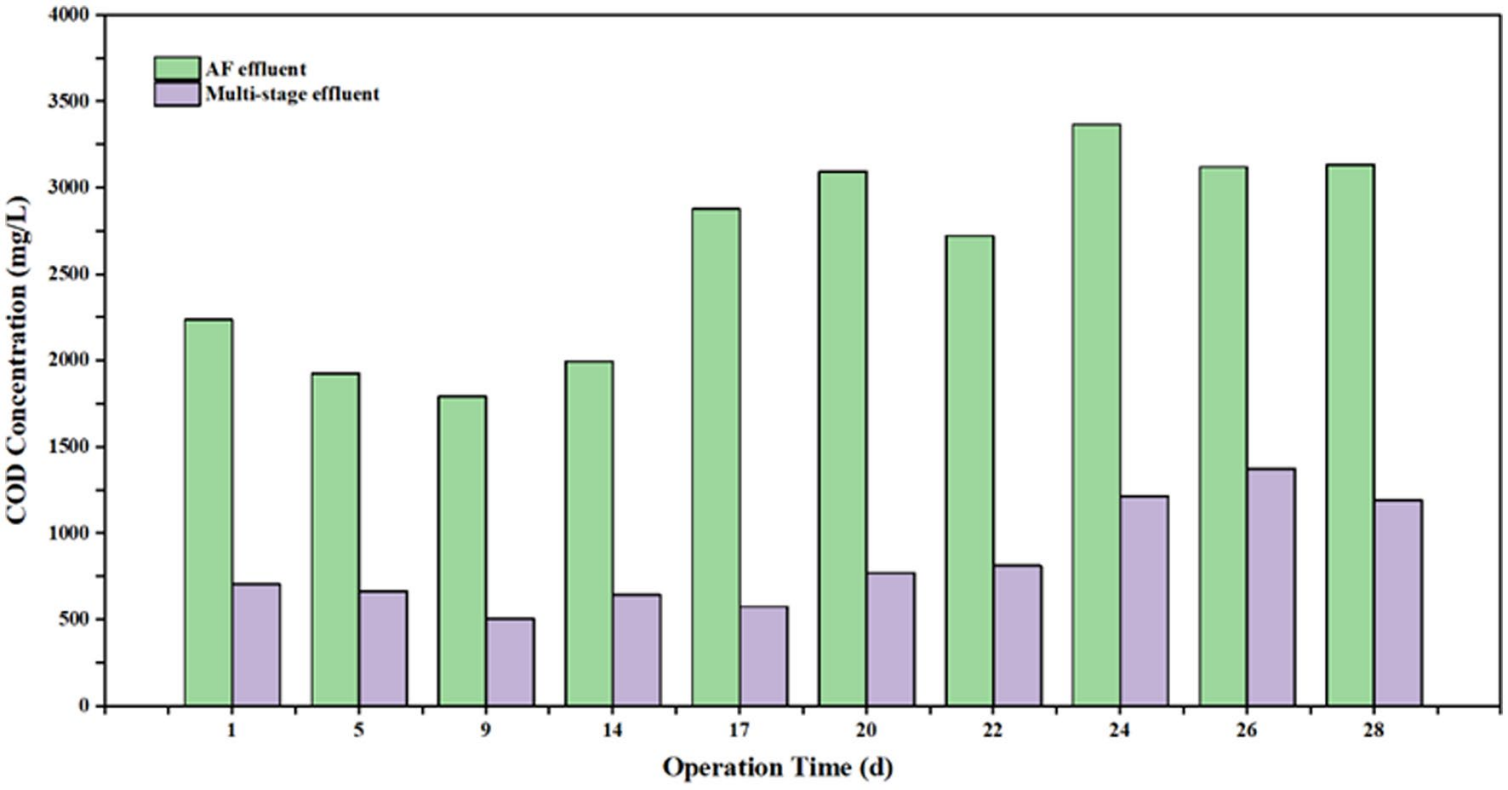
Variations of influent and effluent COD concentrations in the multi-stage biological contact oxidation reactor over the operational period.

**Figure 7:**
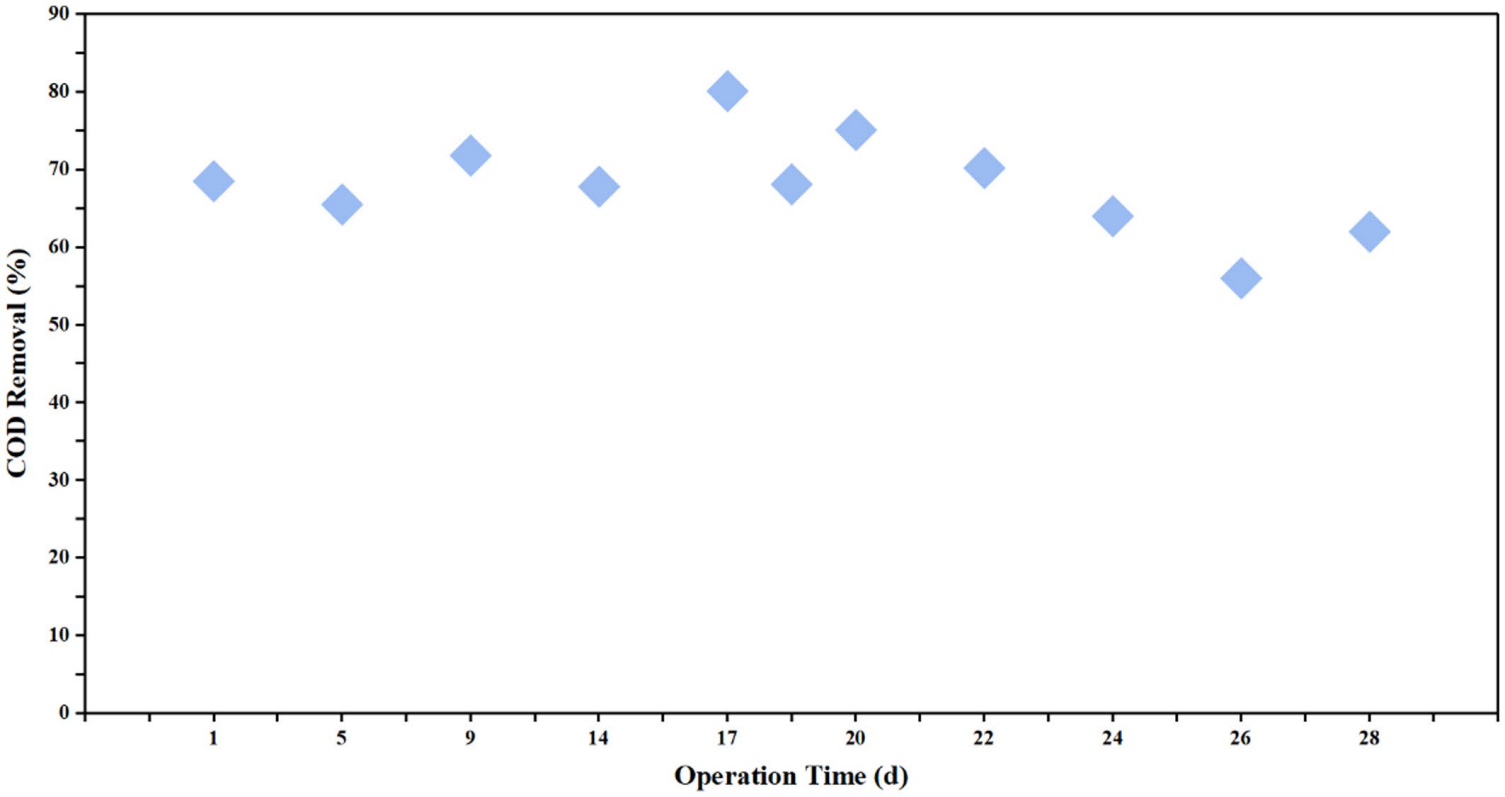
COD removal efficiency of the multi-stage biological contact oxidation reactor during the operational period.

The multi-stage biological contact oxidation reactor demonstrated high removal efficiency even during the initial start-up stage (Figures 6, 7). With the exception of Day 26, the COD removal rate was consistently maintained within the range of 64.1–80.0%. The decline in performance observed on Day 26 was attributed to a surge in the COD concentration of the AF reactor effluent (which served as the influent for the multi-stage reactor). This fluctuation led to an elevated organic loading rate, resulting in increased effluent COD and decreased removal efficiency. For practical engineering applications, it is recommended to increase the hydraulic retention time to buffer against such organic shock loads and ensure stable effluent quality.

On Day 30, the mixed liquor suspended solids (MLSS) concentration in the multi-stage biological contact oxidation reactor was measured at 4151 mg/L, with a SVI of 75.9 mL/g. The high biomass abundance combined with excellent settling properties contributed significantly to the reactor’s high COD removal efficiency.

**Figure 8:**
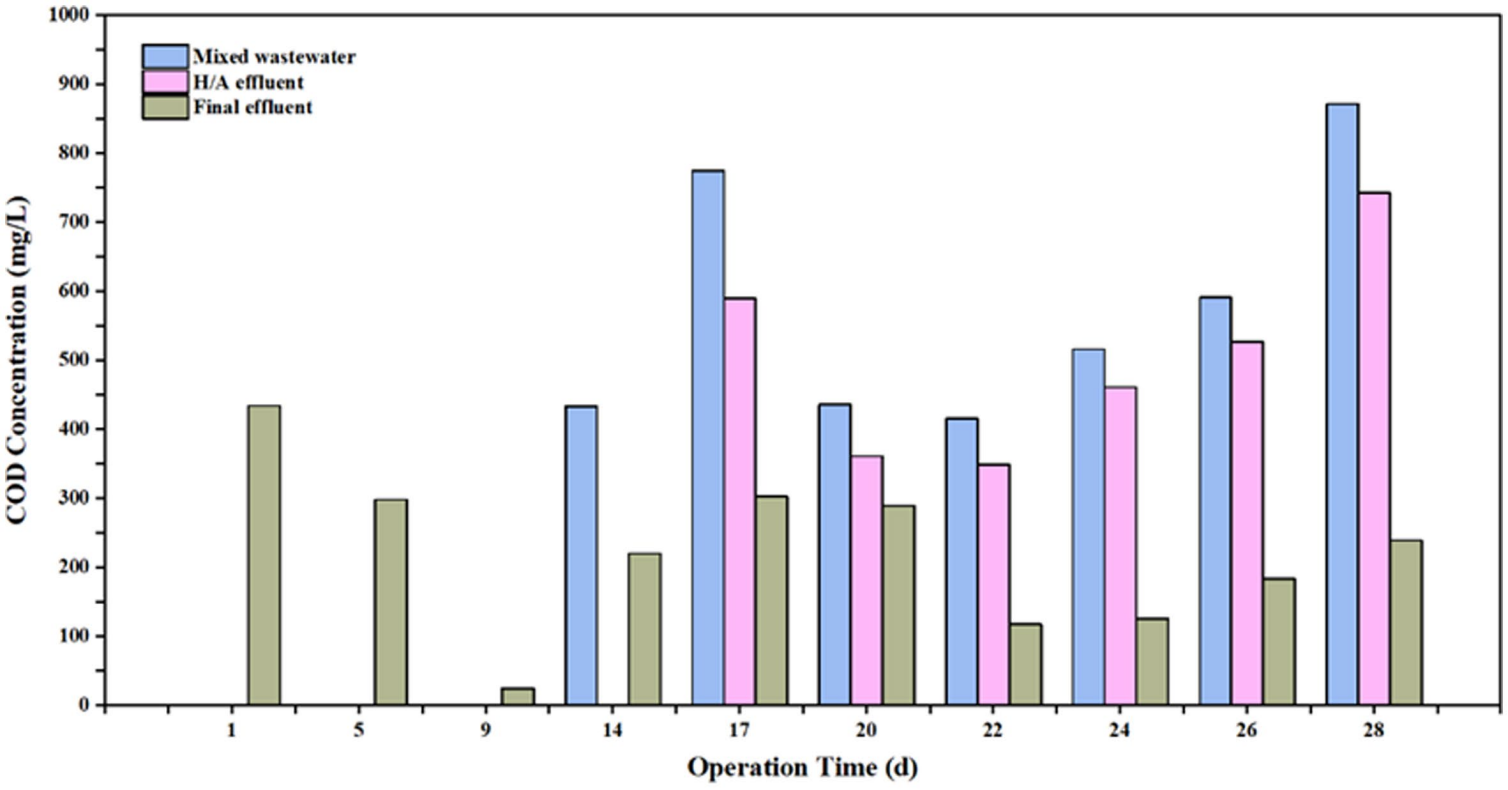
Variations of influent and effluent COD concentrations in the hydrolysis acidification tank and the mixed wastewater contact oxidation tank.

The integrated multi-stage treatment system, comprising the hydrolysis acidification tank, the contact oxidation tank, and the secondary clarifier, achieved stable overall performance (Figure 8). As shown, after the system reached a steady state, the effluent COD concentration was maintained between 117.9 mg/L and 302.3 mg/L, successfully meeting the wastewater treatment requirements.

### 3.4 Performance of the subsequent integrated biological treatment system

The biomass concentrations in the hydrolysis acidification tank and the mixed wastewater contact oxidation tank were determined following the protocols described in Section 2.6, yielding MLSS values of 2,804 mg/L and 5,578 mg/L, respectively.

During steady-state operation, the AF maintained a COD removal efficiency of 20.2–23.5%. However, this performance was found to be temperature-dependent, with an optimal range of 25–35 °C, necessitating thermal insulation in full-scale applications. The multi-stage biological contact oxidation tank achieved a COD removal efficiency of 64.1–80.0%. To mitigate performance declines caused by organic shock loads, extending the hydraulic retention time (HRT) is recommended. The integrated system (hydrolysis acidification/contact oxidation/secondary clarifier) produced a final effluent with COD concentrations ranging from 117.9–302.3 mg/L. The effluent consistently complies with the GB 8978–1996 Grade III limit (500 mg/L), indicating the engineering feasibility of the proposed biochemically driven train for baseline compliance under petrochemical integrated discharge control. However, if a more stringent target is required (e.g., COD ≤120 mg/L under GB 18918–2002 Grade III), the present effluent only approaches or meets this threshold at the lower end of the observed range; therefore, addition routine monitoring would be necessary. If stable compliance with ≤120 mg/L cannot be achieved, supplementary polishing units (e.g., further oxidation/adsorption) may be required, although such options were not experimentally validated in the current work.

Furthermore, the presence of packing media in the contact oxidation tank facilitated the formation of a stable biofilm. The attached microorganisms demonstrated strong resistance to washout under moderate hydraulic shear forces. Consequently, the low concentration of suspended solids in the reactor effluent resulted in minimal sludge accumulation in the secondary clarifier, rendering sludge recirculation unnecessary.

## 4 Discussion

### 4.1 Optimization mechanism and detoxification by coagulation-Fenton pretreatment

This study utilized RSM to confirm that the initial pH is the most critical factor influencing Fenton oxidation efficiency, followed by H_2_O_2_ dosage. A significant decline in COD removal was observed when the pH deviated from the optimal range (2.20–2.23). This aligns with established advanced oxidation theories, which indicate that excess H^+^ scavenges ·OH radicals at extremely low pH, while Fe^2+^ precipitates as Fe(OH)_3_ at higher pH (>3.0), inhibiting the catalytic cycle (Oller et al., 2011). Crucially, targeting the polyether polyols and long-chain polymers prevalent in petrochemical wastewater, the hydroxyl radicals generated by the Fenton reagent non-selectively cleave refractory ether bonds and cyclic structures. Under optimized conditions (pH 2.20, H_2_O_2_ 4.5 mL/L, molar ratio 20), a COD removal efficiency of 51.9% was achieved. This step not only reduced the organic load but also functioned as a vital “detoxification” mechanism, converting macromolecules into smaller volatile fatty acids, thereby overcoming the inhibition to biological systems.

### 4.2 Stability and shock resistance of the biofilm system

Following pretreatment, the MBCO system demonstrated rapid startup and robust degradation performance, maintaining a COD removal efficiency of 64.1–80.0% (except for a short period). Unlike suspended growth systems (e.g., UASB) which are prone to biomass washout, the MBCO system in this study utilized carriers to provide a stable attachment surface, achieving a high Mixed Liquor Suspended Solids (MLSS) concentration of 4,151 mg/L. This biofilm-based structure offers a longer Solids Retention Time (SRT) and stratified microenvironments, facilitating the retention of slow-growing degrading bacteria (Zhou et al., 2021). Previous studies have similarly suggested that staged treatment effectively handles the complex composition of petrochemical effluents. Furthermore, the low SVI value (75.9 mL/g) indicates excellent settling properties, confirming that the biofilm system can maintain microbial community stability under shock loads and sustain biomass balance without the need for sludge recirculation (Ren et al., 2024).

### 4.3 Limitations of environmental factors on system efficacy

Despite the overall excellence of the combined process, the AF unit exhibited significant temperature sensitivity. During periods of ambient temperature fluctuation, its COD removal efficiency dropped sharply from a stable 23.5 to 10.9%. This confirms that the anaerobic hydrolysis process is the rate-limiting step. Anaerobic hydrolytic bacteria are highly sensitive to environmental changes (temperature, pH); a drop in temperature significantly reduces extracellular enzyme activity, thereby hindering the chain scission of long-chain organics (Yang et al., 2015). Therefore, in the AF-MBCO coupled process proposed in this study, strict thermal insulation (maintaining 25–35 °C) for the anaerobic stage is a critical engineering measure to ensure the mechanism of “hydrolysis-acidification improving B/C ratio,” functions effectively, which is vital for securing stable influent quality for the subsequent aerobic stage (Yang et al., 2025).

## 5 Conclusion

To address the challenges posed by the “high-carbon, high-nitrogen, and strong biological toxicity” characteristics of refractory petrochemical wastewater, this study successfully constructed and verified the synergistic purification efficacy of a combined process: “Coagulation-Fenton Oxidation Pretreatment + Biofilm-Driven Multi-Stage Anaerobic-Aerobic (AF-MBCO).” RSM optimization confirmed robust pretreatment parameterization (*p* < 0.05, R^2^ > 0.98), achieving 51.9% COD removal under near-optimal conditions (pH 2.20, H₂O₂ 4.5 mL/L, H₂O₂/Fe^2+^ = 20), close to the predicted optimum (54.4%). At steady operation, overall COD decreased from 3,740 mg/L to 239.2 mg/L (93.6% cumulative removal), with a stabilized final effluent COD of 117.9–302.3 mg/L. Stage-wise performance showed stable AF removal of 20.2–23.5% and consistently high MBCO removal of 64.1–80.0%, supported by high biomass inventory (MLSS 4151 mg/L) and good settleability (SVI 75.9 mL/g). Operationally, AF performance was temperature-sensitive (decreasing from 23.5 to 10.9% during ambient fluctuations), highlighting the need to maintain 25–35 °C for stable upstream conversion, while HRT adjustment provided an effective means to buffer performance under elevated organic loading. Collectively, these results provide quantitative benchmarks and practical operating windows for deploying staged biofilm bioprocesses integrated with chemical pretreatment in refractory petrochemical wastewater treatment.

## Data Availability

The original contributions presented in the study are included in the article/supplementary material, further inquiries can be directed to the corresponding author.
